# Multifactorial analysis and experiments affecting the effect of fog droplet penetration in fruit tree canopies

**DOI:** 10.3389/fpls.2024.1351525

**Published:** 2024-08-08

**Authors:** Daozong Sun, Xinghan Huang, Junyutai Hu, Haoliang Jiang, Shuran Song, Xiuyun Xue

**Affiliations:** ^1^ College of Electronic Engineering (College of Artificial Intelligence), South China Agricultural University, Guangzhou, China; ^2^ Guangzhou Agricultural Information Acquisition and Application Key Laboratory, Guangzhou, China; ^3^ South China Agricultural University, Guangdong Engineering Research Center for Monitoring Agricultural Information, Guangzhou, China

**Keywords:** wind speed, nozzle tilt angle, spraying, droplet size, droplet penetration, plant protection

## Abstract

This study examines the impact of canopy density, side wind speed, nozzle tilt angle, and droplet size on droplet penetration during plant protection spraying operations. Experiments conducted in citrus orchards evaluated how side wind speed and nozzle tilt angle influence droplet penetration across various canopy densities. A Phase Doppler Analyzer (PDA) was used to assess droplet size variations under different nozzle tilt angles and side wind speeds, yielding a multiple linear regression equation (R^2^ = 0.866) that links nozzle tilt angle and side wind speed with droplet size. Results showed that droplet size decreases with increasing nozzle tilt angle at a constant crosswind speed. Further experiments investigated the effects of droplet size and canopy leaf area density on droplet penetration, involving three canopy leaf area densities, four wind speeds, and six nozzle tilt angles. Droplet deposition and canopy coverage were measured under various spraying parameters, with conventional operations (0° nozzle tilt and orthogonal wind speeds) serving as controls. The study found that adjusting nozzle tilt angle and wind speed enhances droplet penetration in different canopy structures. Optimal parameters varied with leaf area density (LAD): an 18° tilt angle and 3 m/s wind speed for a LAD of 5.94 m^3^/m^3^, a 45° tilt angle and 2 m/s wind speed for a LAD of 8.47 m^2^/m^3^, and a 36° tilt angle and 3 m/s wind speed for a LAD of 11.12 m^2^/m^3^. At 1 m/s, droplet deposition followed a downward parabolic trend with changes in nozzle tilt angle, whereas at 2 m/s, deposition followed an upward parabolic trend. At a side wind speed of 3 m/s, droplet deposition remained unchanged with nozzle tilt angle but decreased with increasing canopy density. Nonlinear regression analysis indicated that leaf area density had a greater impact on deposition differences than droplet size, with droplet penetration decreasing as leaf area density increased. This study provides a reference for enhancing fog droplet penetration techniques in plant protection operations, offering practical guidelines for optimizing spraying conditions and improving pesticide use efficiency in different canopy structures.

## 1 Introduction

Effective plant protection spraying is essential for maintaining crop health and maximizing crop yields. The key challenge in this area is to ensure adequate droplet penetration and deposition within the crop canopy. Droplet size is a key factor affecting droplet deposition, penetration, and drift in aerial spraying operations. Smaller droplets are more likely to drift, while larger droplets may not penetrate the canopy effectively. Huynh (Huynh and Nguyen, 2024) and colleagues conducted a comprehensive analysis of the dynamic behavior of droplets in agricultural spraying through modeling and simulation. Their findings revealed that factors such as speed, direction, and evaporation rate significantly influence the efficacy of spraying. By employing precise modeling and simulation techniques, it is possible to predict the behavior of droplets under varying spraying conditions. This allows for the optimization of spray parameters, thereby enhancing the overall spraying effects. Kalyani et al (Kalyani et al., 2023)demonstrated that droplet drift is predominantly influenced by several factors. These include the operational parameters of the spraying equipment, such as nozzle type and spray pressure, environmental conditions like wind speed, temperature, and humidity, and the physical properties of the droplets themselves, such as particle size and density. The importance of optimizing droplet size parameters to improve spraying efficiency and reduce pesticide drift was highlighted in a study by (Chen et al., 2020). It was shown that selecting the appropriate droplet size can significantly improve deposition characteristics and minimize off-target movement, thereby enhancing the overall effectiveness of crop protection. Developing accurate models to predict droplet penetration can help optimize spray parameters. Studies have shown that droplet deposition and penetration depend on the pressure, volume and forward speed of the sprayer, but that these combinations also vary as a function of leaf density (Failla and Romano, 2020; Failla et al., 2020). Duga et al. (Duga et al., 2015) proposed a quadratic exponential regression model based on wind speed, optical porosity, and depth of collection point for predicting droplet penetration. The model provided valuable insights for tuning spray parameters for better penetration in fruit tree canopies, where canopy structure and leaf area density significantly affect droplet behavior. Jomantas et al. (Jomantas et al., 2023) investigated the effect of wind speed on droplet drift and established that an increase in wind speed results in greater droplet drift. Consequently, during spraying operations, it is advisable to select periods with lower wind speeds to minimize droplet drift and enhance penetration effects. Furthermore, research indicates that smaller droplet sizes are more susceptible to wind drift, which can adversely affect penetration effects. Therefore, selecting appropriate nozzles and adjusting spray pressures to control droplet size is a crucial strategy for improving penetration effects. Canopy structure and leaf area density of the crop have a significant effect on droplet penetration. Ru (Ru et al., 2023) pointed out that optical porosity of fruit trees is a key parameter characterizing canopy openness, which is essential for understanding droplet penetration behavior. By measuring optical porosity, the distribution and deposition of droplets in the canopy can be better predicted. Sprinkling parameters such as nozzle type, flow rate, and air volume have significant effects on droplet penetration and deposition. Studies by Cerruto et al. (Cerruto et al., 2021) and Chen et al. (Chen et al., 2023) shown that choosing the right nozzle and adjusting the flow rate can significantly improve droplet penetration in crop canopies. According to Li (Li et al., 2023) it was further shown that air-assisted spraying system could improve droplet penetration and deposition in dense canopies by increasing the droplet kinetic energy.

In addition, a study by Law (Law, 2001) explored the effects of different spraying techniques on droplet penetration. The results showed that the electrostatic spraying technique can significantly improve droplet adhesion and penetration on the crop surface. Electrostatic spraying improves spraying efficiency by electrically charging the droplets so that they are more easily attracted to the crop by the electric field. Shi et al. (Shi et al., 2022) also pointed out that environmental conditions such as wind speed, humidity, and temperature have important effects on droplet penetration and deposition. High wind speed may lead to droplet drift, while high humidity and suitable temperature help droplet attachment and penetration on the crop surface. Therefore, environmental conditions need to be considered comprehensively to optimize the spraying effect in actual spraying operations. For conventional boom sprayers, which are widely used in field crops, the penetration effect can only be improved by changing parameters such as spray pressure, spray flow rate, and nozzle type, but this will also have an impact on the spraying effect (Sun et al., 2021). The droplet deposition effect can be effectively improved by changing the nozzle tilt angle (Zhu et al., 2004; Ferguson et al., 2016; Legleiter and Johnson, 2016); but in the current research on adjusting the nozzle tilt angle, the nozzle tilt angle and deposition amount of vertical spray were mostly analyzed. In contrast, there are fewer studies exploring the effects of different side winds and different nozzle tilt angles on the effect of droplet penetration in the horizontal spraying method, which is common in plant protection operations for fruit trees.

This paper investigates the influence of various parameters, including canopy density, sidewind speed, nozzle tilt angle, and droplet size, on the penetration effect of fog droplets in plant protection spraying operations. The study decomposes these factors into two parts for experimental analysis: firstly, the impact of the nozzle tilt angle and sidewind speed on fog droplet size; secondly, the influence of fog droplet size and canopy leaf area density on spray penetration. The objective is to identify optimal spraying conditions that enhance the distribution and deposition of fog droplets within tree canopies and to establish related rules. The research focuses on the sidewind speed and nozzle tilt angle (with the direction of the sidewind perpendicular to the nozzle direction), examining their effects on the variation in fog droplet size under different leaf area density canopies. Adjustable constant speed wind in the experimental wind field simulates varying sidewinds to affect fog droplet size. By modifying the tilt angle of the fog droplet nozzle, the study explores the relationship between fog droplet size and deposition differences under orthogonal sidewind and nozzle tilt angle. Multivariate nonlinear regression analysis technology, combined with spraying experiments, systematically evaluates the effects of sidewind speed and nozzle tilt angle on fog droplet penetration. This research provides a reference for optimizing the operating parameters of pesticide application equipment, achieving precise pesticide application, and enhancing the prevention and control of fruit tree diseases and pests.

## 2 Materials and methods

### 2.1 Test materials

**Figure 1:**
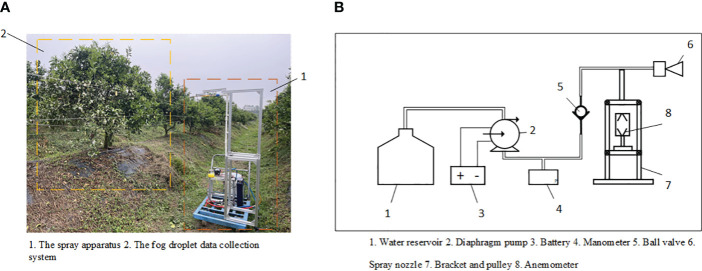
**(A)** Test site. **(B)** Structure of the spraying device.

The system used for the spray test consists primarily of two components, as illustrated in **Figure 1A**: the spray device and the droplet information acquisition system. The physical representation of the spraying device is labeled as 1 in **Figure 1A**, and its corresponding structure is shown in **Figure 1B**. The spraying device includes a water tank, a diaphragm pump (DP-160, flow rate 7L/min, pressure 0.3Mpa), a spray nozzle (JJXP type, solid conical, rated pressure 0.3Mpa), and an anemometer (WindMaster Pro, measuring three-dimensional wind speed and direction from 0 to 65 m/s).

**Figure 2:**
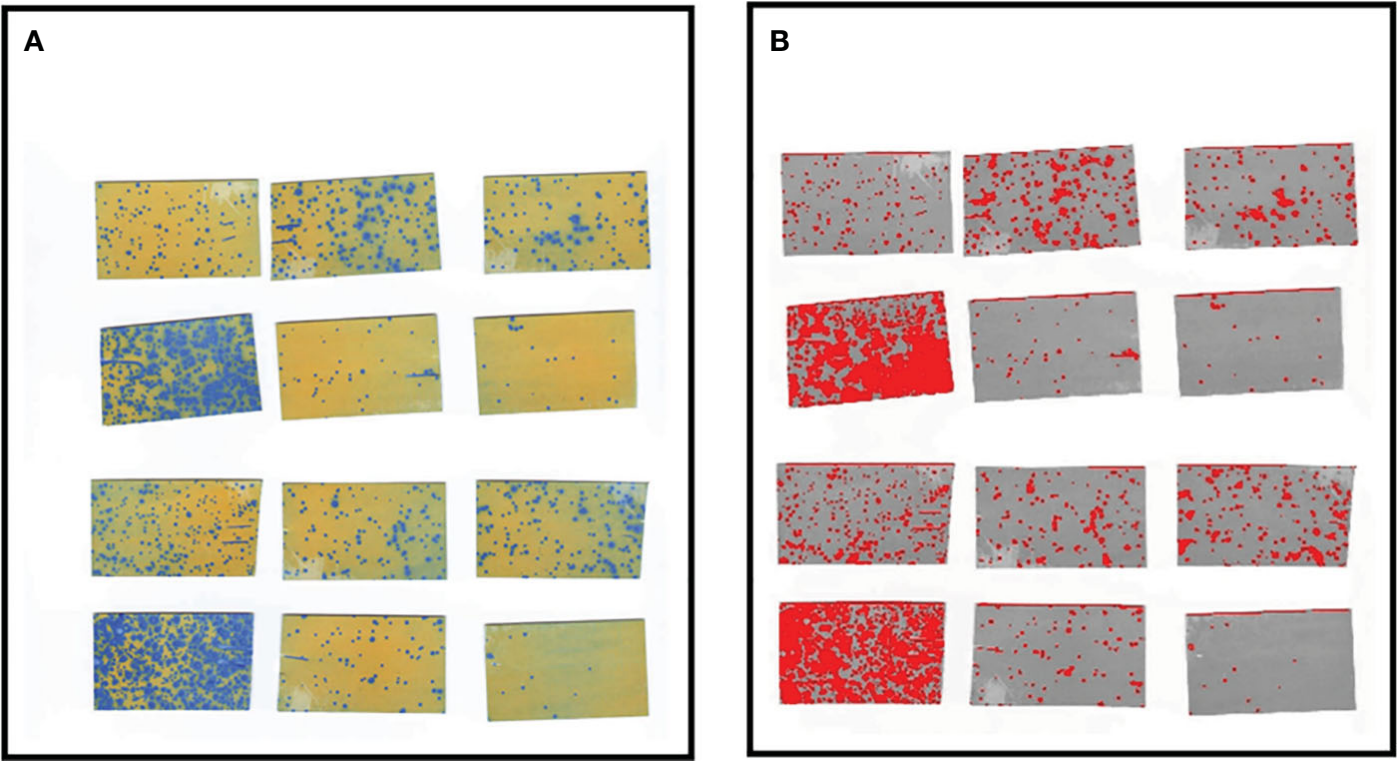
**(A)** Sample point scan image. **(B)** Processed images.

The droplet information acquisition system, labeled as 2 in **Figure 1A**, consists of three layers of capillary line groups (with intervals of 30-35 cm between the front and back capillary lines and 30 cm between the top and bottom layers), a test tree, and water-sensitive paper (76 x 26 mm) used to collect fog droplet information, part of which is shown in **Figure 2**. The test tree specifications are 180 cm in height, 150 cm in crown width, and leaf area densities of 5.94, 8.47, and 11.12 m²/m³, with a tree spacing of 150 cm arranged in a north-south direction. The study primarily investigated the effect of canopy density on fog droplet penetration under different side wind speeds, using manual methods to measure canopy leaf area density as a reference value.

According to Sun et al. (Sun and Liu, 2019), the branching coefficient case of the canopy uses the leaf area density ρ, which refers to the sum of leaf areas per unit volume, as shown in Equation 1:


(1) $$\rho =\frac{S}{V}$$

Where S is the leaf area per unit volume, m^2^; V is the canopy volume containing that total number of leaves, m^3^.

Assuming that the number of leaves in the area volume V is $n_{l}$ , The surface area of each leaf is $s{\;}_{i}$ ( i = 1, 2, ..., n *_l_* ), Sort the leaves by number, and the difference between the leaf area of each leaf and that of the first leaf is denoted as $\text{Δ}s{\;}_{i}$ ( i = 1, 2, ..., n *_l_* — 1), Then the leaf area within the volume of the region V can be expressed as shown in Equation 2:


(2) $$s=s_{1}n_{l}+\sum_{i=1}^{n_{l}-1}\text{Δ}s_{i}$$

Assuming that $s_{1}n_{l}\gg \sum_{i=1}^{n_{l}-1}\text{Δ}s_{i}$ , i.e., the change of each leaf area within the regional volume V is relatively small, the leaf area within the regional volume V is simplified as $s=s_{1}n_{l}$ , which can be obtained by substituting it into Equation 3:


(3) $$\rho =\frac{s_{1}n_{l}}{V}$$

Where ρ is the leaf area density in the region, m^2^/m^3^.

**Figure 3:**
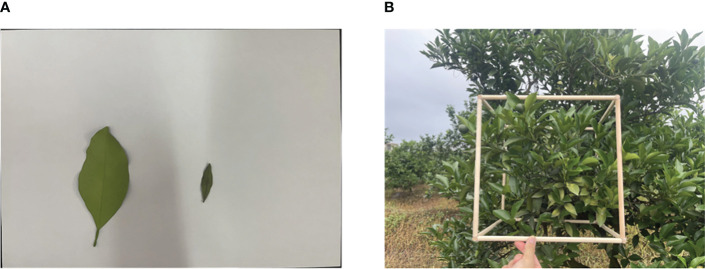
**(A)** The measurement of average leaf area; **(B)** The measurement of canopy density data.

Measurements were conducted using 30* 30 *30cm square sample frames, as shown in **Figure 3**. Different areas of the canopy were randomly selected for measurement. The number of leaves within each sample frame was counted and recorded. A certain number of leaves were then randomly selected from each sample frame, and their areas were calculated using image processing and other methods. The mean leaf area from the samples was used to determine the overall mean leaf area within each sample frame. This value was then used to calculate the leaf area bulk density of the sample frame, which represents the leaf area bulk density of that specific canopy partition, also referred to as the thinning density of the canopy.

### 2.2 Experimental design and methods

To investigate the effects of canopy density, side wind speed, nozzle tilt angle, and droplet particle size on droplet penetration inside the canopy, the experiment was divided into two parts. In part one, Phase Doppler Anemometry (PDA, Dantec Dynamics A/S, Denmark) was used to study the variation patterns of droplet size under different wind speed levels and nozzle tilt angle parameters orthogonal to the spray nozzle (Durdina et al., 2012). In part two, water-sensitive paper collected droplet deposition data under various spray parameters, and DepositScan imaging software analyzed droplet deposition coverage on each layer of the target, as well as differences in droplet deposition at each canopy leaf area density.

#### 2.2.1 Droplet size distribution test

**Figure 4:**
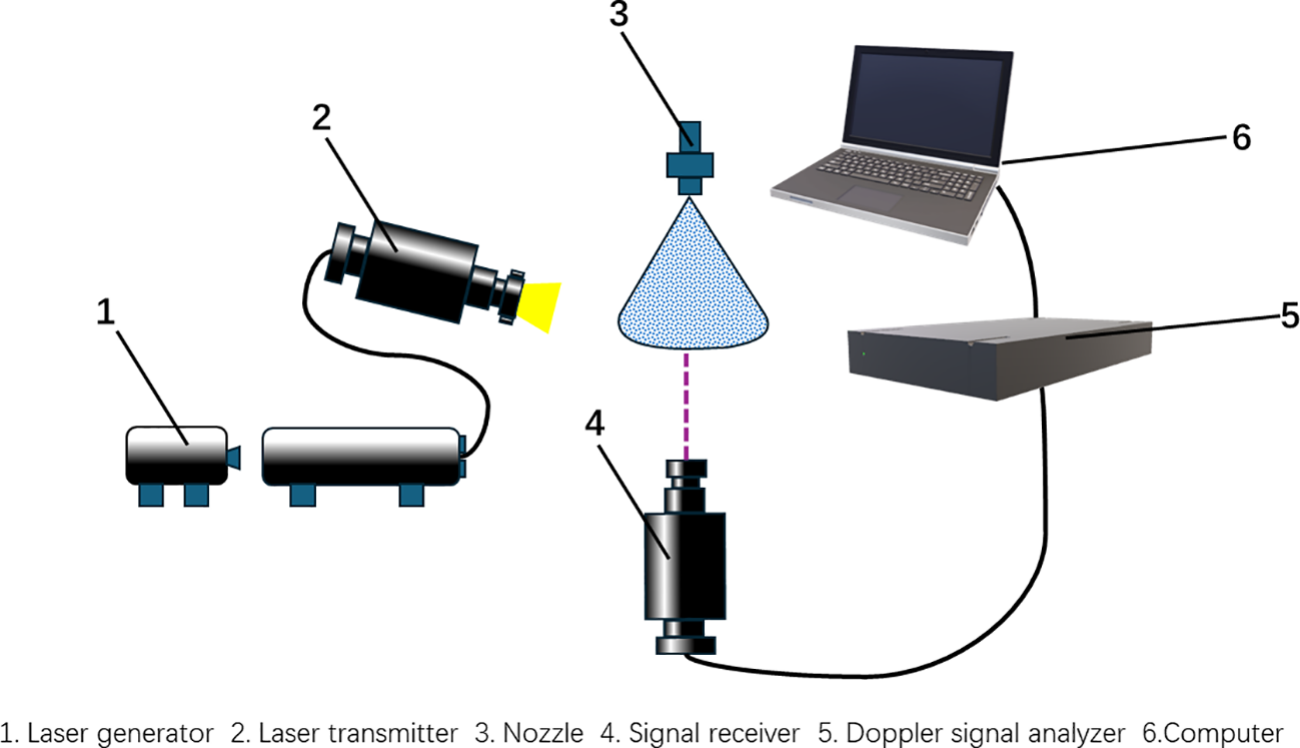
Schematic diagram of particle size distribution measurement device.

**Figure 5:**
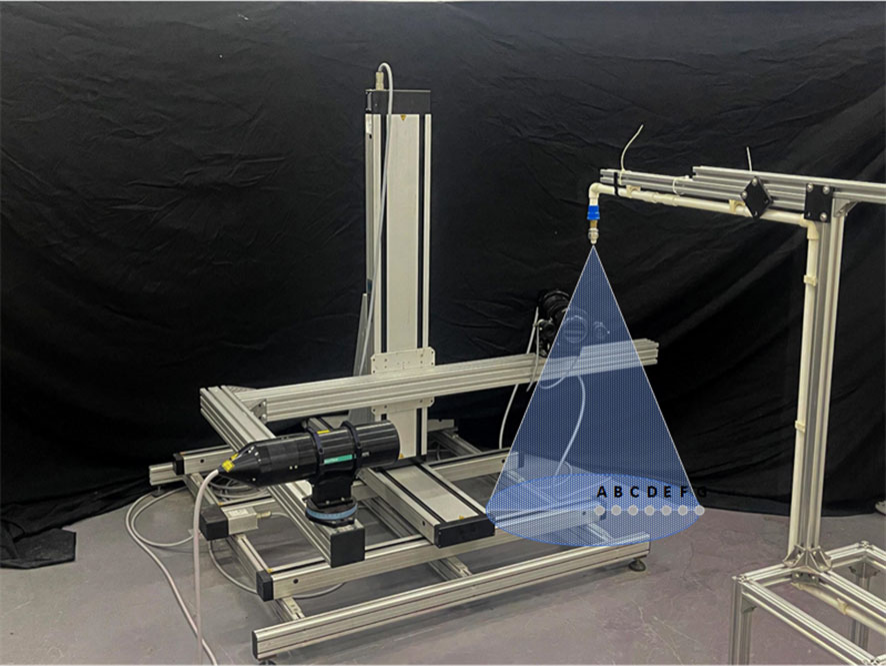
Particle size distribution measurement site.

The spray nozzle was fixed to the spray frame and mounted on a universal spray head. The experiment was designed with four wind speed levels (0, 1, 2, and 3 m/s) and seven nozzle tilt angles (0°, 9°, 18°, 27°, 36°, 45°) for droplet size measurement. An aluminum frame with dimensions of 2.5 * 0.7 * 1.6 m was placed underneath the nozzle. The distance between the spray nozzle and the laser transmitter was adjusted to 1 m, which is the typical distance between a sprayer and fruit trees during plant protection spraying operations. The Phase Doppler Anemometry (PDA) system performed single-point measurements, with the spraying center axis 1 m from the spray nozzle as the origin. Measurement points were spaced at 5 cm intervals on the horizontal plane. The device structure is shown in **Figure 4**, and the sampling scene is shown in **Figure 5**. Each plane was divided by the radius formed by straight lines connecting the measurement points to the origin, with a spacing of 5 cm, totaling six sampling points labeled A to F from inside to outside.

The PDA used laser light at wavelengths of 514.5 nm (green) and 488 nm (blue), with a focal length of 800 mm between the transmitting and receiving probes and a scattering angle of 67°. The sampling condition required either reaching a data volume of 100 at the measurement point or a measurement time of 10 seconds. If either condition was met, the system automatically moved to the next measurement point.

According to Xue (Xue et al., 2022) and others, the droplet size distribution or droplet mean diameter is usually used to evaluate the atomization quality and characteristics. It is sufficient to use the mean droplet diameter for general studies, and although the volume median diameter or the number median diameter are characteristic diameters, they do not fully reflect the atomization quality. Sauter Mean Diameter (SMD, D32) characterizes the mass and surface area of the droplet population and can reflect the basic characteristics of similar systems.

It is defined as (Equation 4):


(4) $$D_{32}=\frac{\sum_{i-1}^{N}D_{i}^{3}}{\sum_{i-1}^{N}D_{i}^{2}}$$

Where $D_{i}$ is the diameter of the ith droplet, μm; *N* is the number of droplets.

The results of the Doppler tester measurements on the JJXP nozzle will summarize its droplet solt mean diameter and droplet velocity distribution law for this nozzle under different nozzle tilt angles and crosswind wind speeds.

#### 2.2.2 Droplet deposition difference test

To explore the effect of spray nozzle tilt angle on the penetration of fog droplets in canopies with different leaf area densities under varying crosswind speeds, the experimental conditions were set as follows: the nozzle height was 20 cm from the ground, the spray pressure was 0.3 MPa, wind speeds were 0, 1, 2, and 3 m/s, and nozzle tilt angles were 0°, 9°, 18°, 27°, 36°, and 45°. The control conditions were no wind (0 m/s) and a nozzle tilt angle of 0°.

**Figure 6:**
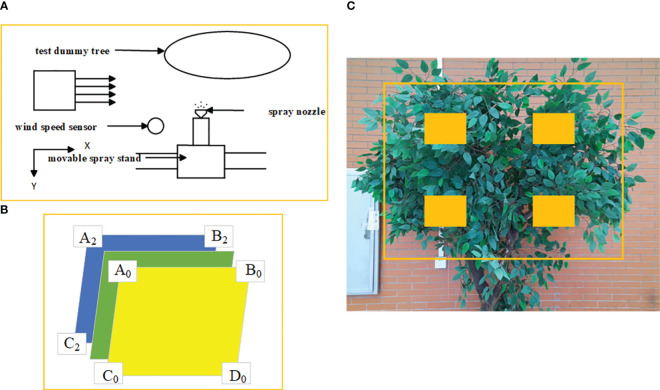
**(A)** Structure of droplet penetration test; **(B)** Schematic diagram of the location of measurement points; **(C)** Sampling point layout.

The experimental setup, shown in **Figure 6A**, had the nozzle fixed with its initial direction perpendicular to the test tree, and the wind direction orthogonal to the spray direction. The anemometer was positioned 50 cm from the nozzle in the X direction, and the test tree was 100 cm from the spraying device. Water was used instead of pesticide in the experiment. The tree canopy was divided into three layers, with four fog volume collection points (Ai, Bi, Ci, Di) in each layer.

The measurement of droplet deposition involved the following steps: First, as shown in **Figure 6C**, the canopy was divided into three layers from front to back along the spray direction. Each layer was fixed to neighboring citrus trees on the left and right sides of the test tree with thin lines connected in a straight line through the canopy. Four measurement points (Ai, Bi, Ci, and Di) were uniformly arranged on the plane formed by the two thin lines. Water-sensitive paper was fixed at the measurement points with paper clips, and the collection surface was perpendicular to the spray direction. The position of the water-sensitive paper and thin lines was adjusted to avoid shading. The spraying operation was then carried out for one second. After drying, the water-sensitive paper was collected, and the experiments were repeated three times, with the average value taken. The experiment was repeated with varying wind speeds and spray nozzle tilt angles for multiple measurements. Finally, droplet deposition was analyzed using DepositScan software, and the differences in droplet deposition in each canopy layer were calculated using Equations 5, 6.

Droplet penetration distribution was evaluated using the difference in droplet penetration deposition (Wang, 2002), where penetration deposition indicates the deposition of droplets in the depth direction of the canopy, and droplet deposition in each vertical plane was indicated by the mean droplet deposition, and the confidence interval for the mean deposition was calculated to reflect the degree of variability of the sampling points in that vertical plane. In the test analysis, the change in deposition value was used to represent the change in deposition in the test group compared to the control group. The formula for calculating the average deposition is (Equation 5):


(5) $$Q_{i}=\frac{1}{4}\sum_{j=1}^{4}q_{ij}$$

where i is the fog droplet distribution layer position, in this experiment, a total of three layers were set up (as shown in **Figure 6B**), that is, i = 0, 1, 2, i is 0 means in the outermost layer; j is the position of each fog droplet capture point on the capture surface of each layer, according to the order from top to bottom from left to right in order numbered from 1 to 4, with a total of 4 capture points, j = 0~4; qij is the amount of fog droplet deposition on the water-sensitive paper on the capture point, which was analyzed by the DepositScan software scanned and analyzed.

The formula for the difference in deposition is (Equation 6):


(6) $$D_{i}^{vn}=Q_{i}^{vn}-Q_{i}^{v0}$$

Where, v is the wind speed value, v=0, 1, 2, 3; n - nozzle tilt angle, n=0, 9, 18, 27, 36, 45. i is the droplet distribution stratum, i=1, 2; Q_i_ is the average volume of fog per unit of vertical surface within the canopy corresponding to stratum i, μL/cm²; Q_0_ is the average volume of fog per unit of vertical surface at the edge of the canopy just before entering the canopy, μL/cm;

The confidence interval is calculated as (Equation 7):


(7) $$CI=\bar{x}\pm (t_{\frac{\alpha }{2},df}\times \frac{s}{\sqrt{n}})$$

where CI is the confidence interval; $\bar{x}$ is the sample mean; ${\text{t}}_{\frac{\text{α}}{2},\text{df}}$ is the t-value of the t-distribution, which corresponds to the chosen confidence level and degrees of freedom; the 95% confidence level was chosen in the paper; s is the sample standard deviation; and n is the sample size.

## 3 Experimental results and analysis

The experiment was conducted using the controlled variable method, with the spray test site on the sixth floor overhead of the North Building of the College of Engineering, South China Agricultural University, Guangdong Province, China, in May 2024, and the field test site at the base of Great Orange Orchard Pingtan, Pingtan Town, Huidong County, Huizhou City, Guangdong Province, China, in June 2024, with an average ambient temperature of 29°C and an average ambient humidity of 56% at the time of the test.

### 3.1 Spray test results and analysis

#### 3.1.1 Spraying test results and analysis

Multiple nonlinear regression was performed with wind speed and tilt angle as independent variables and mean particle size of fog droplets as dependent variable, and the regression equation is shown in Equation 8.


(8) $$P=av^{2}+bn^{2}+cvn+dv+en+f$$

Where P is the average particle size of the droplets, μm; v is the side wind speed, m/s; n is the nozzle tilt angle, °.

**Figure 7:**
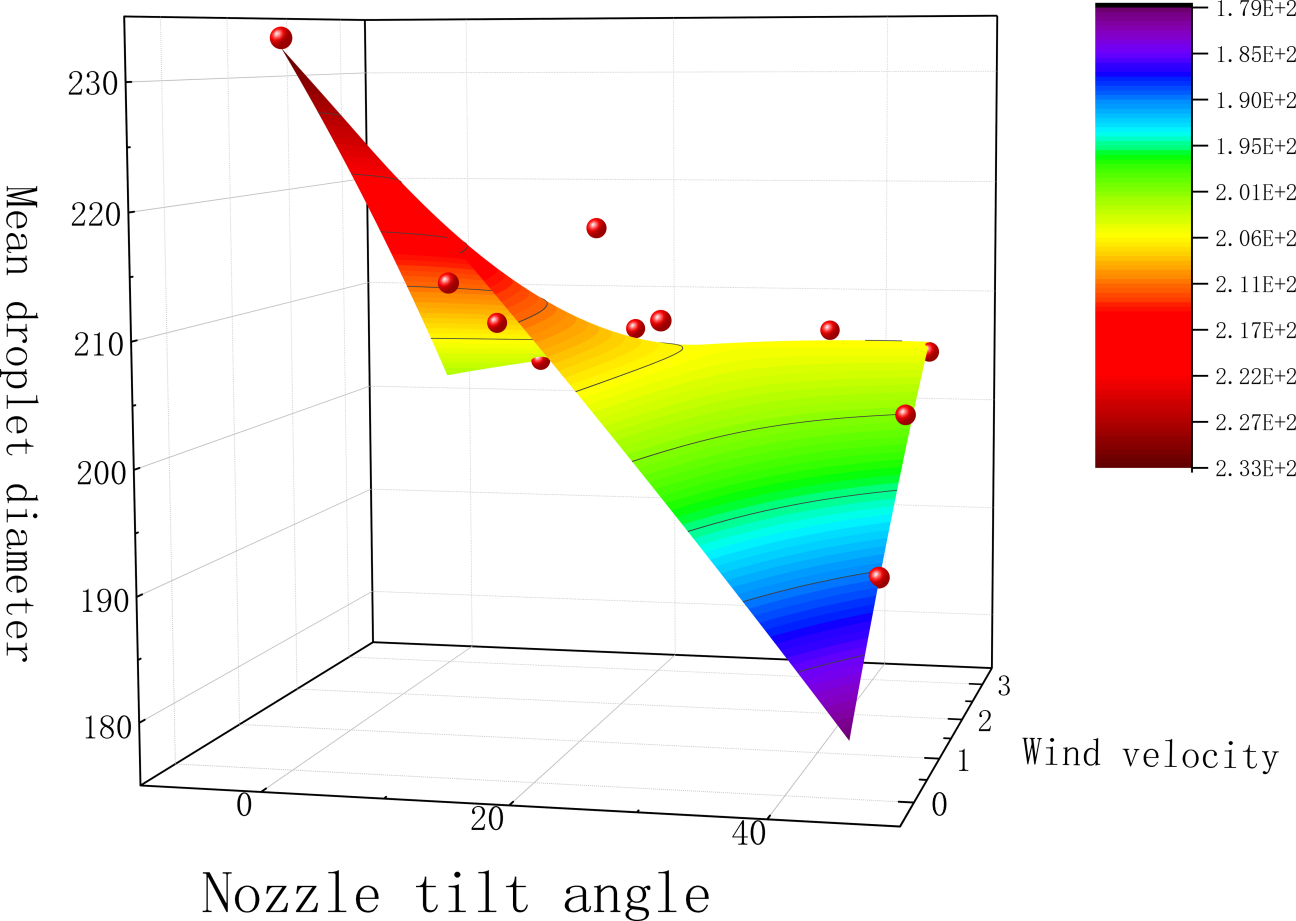
Mean droplet diameter for different parameters.

The fitting results are shown in **Figure 7** and **Table 1**. The R² value of the regression model is 0.8662, indicating a good fit and correlation. The coefficient of wind speed (v) is less than that of the tilt angle (n), suggesting that the tilt angle has a greater effect on the average droplet size than wind speed. The interaction term (vn) in the model describes the combined effect of wind speed and nozzle tilt angle, with positive coefficients indicating that increasing the tilt angle enhances the average droplet size as wind speed increases. The minimum tilt angles of 45°, 36°, and 27° were calculated for the average droplet size under wind speeds of 1, 2, and 3 m/s, respectively.

**Table 1:** The values of the fitted parameters.

parameters	numerical value
a	-2.37E-3 ± 7.48E-3
b	-4.83E-1± 1.74
c	4.21E-1 ± 9.26E-2
d	-1.07 ± 3.99E-1
e	-8.71 ± 5.91
f	232.52 ± 2.92
R^2^	0.866

As shown in **Figure 7**, the droplet size distribution under three different wind speeds generally decreases with increasing tilt angle. The maximum droplet size at 1 m/s occurs at a 9° tilt angle, at 2 m/s at an 18° tilt angle, and at 3 m/s at an 18° tilt angle. The highest recorded droplet size was at a wind speed of 2 m/s and a tilt angle of 18°. Three groups of test droplet sizes closely matched the control group, with a maximum variation of 23%, indicating that changing the nozzle tilt angle under different wind speeds affects the droplet size to varying degrees.

At a tilt angle of 0° and a wind speed of 0 m/s, the droplets did not undergo secondary fragmentation, resulting in larger and more stable droplets that were less prone to drift. However, droplets with a larger average diameter were more easily blocked by leaves and had difficulty penetrating denser canopies. At a tilt angle of 9°, droplet size decreased with increasing wind speed. For tilt angles greater than 9°, droplet size increased with wind speed. The decrease in droplet size may be due to several factors: 1) Side winds increase the frequency and intensity of collisions between droplets, causing them to break into smaller particles. 2) As the nozzle tilt angle increases, the spray path of the droplets becomes more complex and dispersed. 3) A larger tilt angle extends the droplets’ flight path, making them more susceptible to wind speed and fragmentation. The reason for the increase in droplet particle size may be: 1) According to Wang et al. (Wang et al., 2024) Under low-pressure spraying conditions, the increase in wind speed will make the fine droplets able to merge and aggregate more effectively under the action of wind, which will make the average particle size of the droplets larger and the droplet spectral width smaller accordingly.

#### 3.1.2 Distribution of droplet penetration deposits

**Figure 8:**
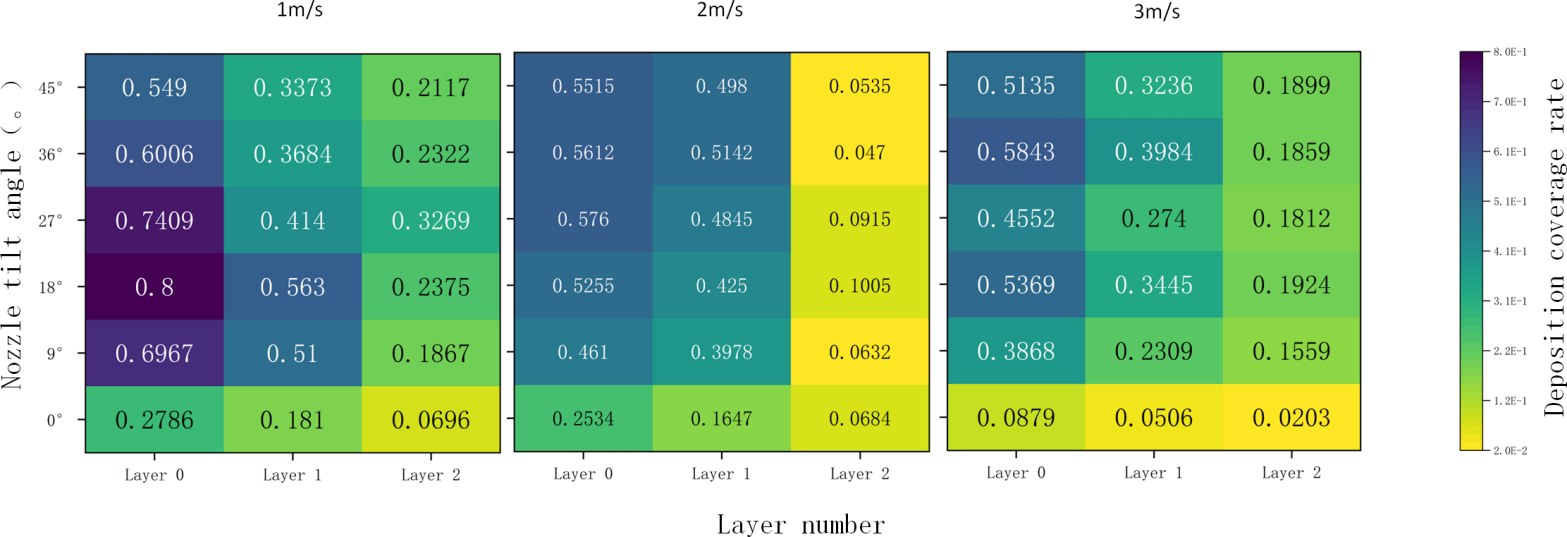
Average deposition on each layer for different parameters.

**Figure 8** compares the deposition volume at different canopy leaf area densities and wind speeds for each inclination angle. The deposition volume is averaged across all deposition data for each layer, with 95% confidence intervals represented as error bars. It is observed that the amount of fog droplets deposited in layer 0 is significantly larger than in layers 1 and 2. The data show that fog droplets are attenuated by nearly 30% after the first canopy layer and by nearly 75% after the second layer. This significant attenuation is due to shading by branches and leaves, as well as gravitational effects, causing most fog droplets to be deposited on the canopy surface.

At a low wind speed (1 m/s), the deposition in the outermost layer (layer 0) increases and then decreases with the nozzle tilt angle, exhibiting a peak value. This suggests an optimal nozzle tilt angle that maximizes deposition in the outer layers of the canopy. The deposition in the middle (layer 1) and inner (layer 2) layers also fluctuates with changes in the nozzle tilt angle, but the pattern is less pronounced than in the outer layer. Observing the droplet deposition distribution in layer 0 shows that deposition in layers 1 and 2 increases and then decreases with increasing tilt angle, while deposition in layer 3 exhibits a fluctuating trend. The maximum deposition values for layers 1 and 2 occur at 18° and 27° inclination angles, respectively.

The observed distribution patterns can be explained as follows: At a wind speed of 1 m/s, droplet drift is minimal, allowing most droplets to be captured by the target, and the tilt angle has less impact. At 2 m/s, droplet drift is more pronounced, and changes in the tilt angle help compensate for this drift, significantly affecting droplet deposition. At a wind speed of 3 m/s and tilt angles greater than 30°, the initial direction of the droplets in the nozzle becomes crucial in determining their interaction with the wind velocity.

When the wind speed was 3 m/s and the tilt angle exceeded 30°, the angle between the initial direction of the spray nozzle droplets and the wind field velocity significantly impacted droplet fragmentation. Larger droplets fragmented into smaller ones, increasing droplet drift, and the drift effect outweighed the compensatory effect (Sun et al., 2021), resulting in decreased droplet deposition. Observations of droplet distribution within the canopy showed that the deposition amount generally increased and then decreased, with most deposition concentrated between 18° and 36°, peaking at 27° and lowest at 45°. This pattern suggests that changes in spray tilt angle reduced the initial droplet velocity at 0°, thereby decreasing the droplet penetration distance. Consequently, most droplets lacked sufficient velocity to reach the second layer.

The error bars indicate greater variability in deposition on the outside of the canopy and under conditions with smaller tilt angles. This variability may be due to the inhomogeneity of droplet deposition influenced by side winds or inherent measurement errors, as shown in **Figure 8**. Lower variability in deposition volume within the inner canopy and under conditions with larger tilt angles may be attributed to the overall lower deposition volume inside the canopy.

In summary, the average deposition amount of each layer in the test group was larger than the droplet deposition amount in the control group, indicating that wind speed significantly affected the deposition amount of fog droplets (Wang et al., 2016), and when there was a side wind or the tilt angle was shifted, on the one hand, the fog droplets were subjected to air resistance in the horizontal direction, which changed the trajectory of the fog droplets, and some of them produced drifting away from the target (Zhang et al., 2017). On the other hand, in the horizontal direction, the initial motion direction of the fog droplets is opposite to the direction of the crosswind, resulting in the intensification of the mutual motion of the fog droplet group, and more phenomena such as polymerization, splitting, and secondary splitting occur (Shen et al., 2022), and more large droplets break up into small droplets, which makes it difficult for small droplets to reach the target mark under the action of the air resistance and other factors.

The deposition amount under the same wind speed and different tilt angles was compared with that at a 0° tilt angle to analyze the effect of changing the nozzle tilt angle on droplet deposition under the same side wind. **Figure 8** shows the changes in deposition volume on each vertical surface under different parameters. It is evident that, in most cases, the deposition volume at a 0° tilt angle is smaller than at other tilt angles for the same wind speed. **Figure 8** also shows that there are almost no droplets inside the canopy at 0° when the wind speeds are 2 m/s and 3 m/s, whereas droplets are captured at other tilt angles. This indicates that adjusting the tilt angle can improve droplet deposition inside the canopy.

The size relationship of the data in the horizontal view generally follows the pattern P0 > P1 > P2, indicating that the impact of changing the tilt angle on deposition decreases with the depth of the canopy. The deeper the canopy, the smaller the change in deposition amount. In summary, compared with the control group, changing the tilt angle can effectively increase overall canopy deposition, particularly in the outermost layer, and enhance deposition within the inner canopy, thereby improving droplet penetration.

The differences in deposition amounts for various canopies were summarized as the overall change in deposition under different conditions. The average change in deposition under different lateral winds at the same tilt angle represented the difference in deposition at that angle. The differences in deposition amounts at tilt angles of 9°, 18°, 27°, 36°, and 45° were calculated to be 40.98%, 40.19%, 40.87%, 42.76%, and 39.72%, respectively. This indicates that under the same wind speed, proper adjustment of the nozzle tilt angle in the opposite direction of the side wind can effectively increase droplet deposition in the canopy. This is likely because, at a 0° tilt angle, the side wind alters the droplet trajectory, causing most droplets to miss the target, whereas adjusting the nozzle tilt angle compensates for droplet drift (Sun et al., 2021).

Multiple nonlinear regression was performed with droplet particle size and canopy leaf area density as independent variables and difference in deposition as dependent variable, and the regression equation is shown in Equation 9.


(9) $$P=aD^{2}+bS^{2}+cDS+dD+eS+f$$

where P is the difference in droplet deposition, %; D is the average droplet particle size, μm; and S is the canopy leaf area density, m^2^/m^3^.

**Figure 9:**
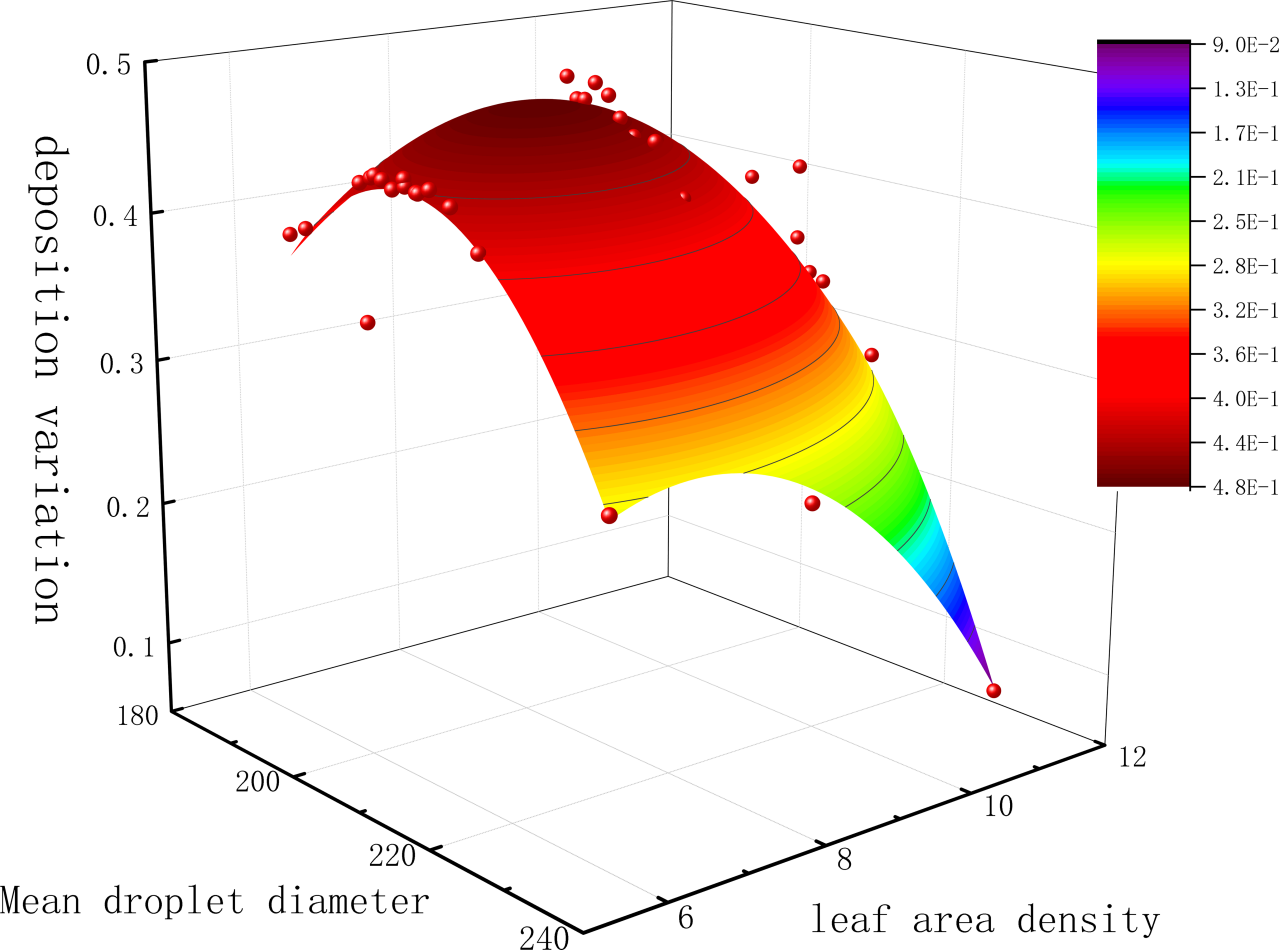
Multivariate nonlinear surface fitting graph.

The fitting results are shown in **Figure 9** and **Table 2**. The R² value of this regression model is 0.9047, indicating a good fit and strong correlation. The coefficient of droplet particle size (D) is smaller than that of canopy leaf area density (S), suggesting that canopy leaf area density has a greater impact on deposition differences than droplet particle size. The interaction term (DS) describes the combined effect of wind speed and sprinkler tilt angle, with negative coefficients indicating that as the average droplet particle size increases, the increase in canopy leaf area density reduces deposition differences. The distribution patterns of horizontal droplet deposition differences were calculated for leaf area densities of 5.94, 8.47, and 11.12 m²/m³.

**Table 2:** The values of the fitted parameters.

parameters	numerical value
a	-2.25E-4 ± 2.20E-5
b	-0.01.16E-2 ± 1.11E-3
c	-6.47E-4 ± 1.72E-4
d	9.67E-2 ± 9.38E-3
e	3.13E-1 ± 4.02E-2
f	-1.06E1 ± 1.02
R^2^	0.905

At a tilt angle of 0°, fog droplets drifted under the influence of the side wind, preventing most droplets from reaching the target and reducing penetration effectiveness as wind speed increased. As shown in **Figure 9**, the variation in fog droplet deposition amounts under different wind speeds shows a similar trend. At a crosswind speed of 1 m/s, the deposition difference with increasing nozzle tilt angle exhibits a downward-opening parabolic trend. The maximum deposition difference occurs at different tilt angles for varying canopy densities: at a leaf area density of 5.94 m²/m³, the maximum deposition difference occurs at a 9° tilt angle (0.436); at 8.47 m²/m³, it occurs at an 18° tilt angle (0.46); and at 11.12 m²/m³, it occurs at a 36° tilt angle (0.34).

The reason for the increase in the effect of fog droplet penetration could be: 1) Higher canopy leaf area density creates a more complex network of obstacles. Increasing the nozzle tilt angle can alter the droplet movement path, increasing the contact angle with leaves, which helps droplets penetrate better by reducing surface layer contact (Ma et al., 2022). 2) At higher tilt angles, the spray becomes more parallel to the leaf surface, reducing direct impact and rebound, thereby enhancing penetration into the leaf surface and canopy gaps. 3) In high-density canopies, vertical spray tends to accumulate on upper leaf surfaces, forming a “surface barrier” that hinders subsequent droplet penetration. A larger nozzle tilt angle helps droplets slide over surface leaves, reducing surface accumulation and promoting more uniform spray distribution and deeper penetration.

**Figure 10:**
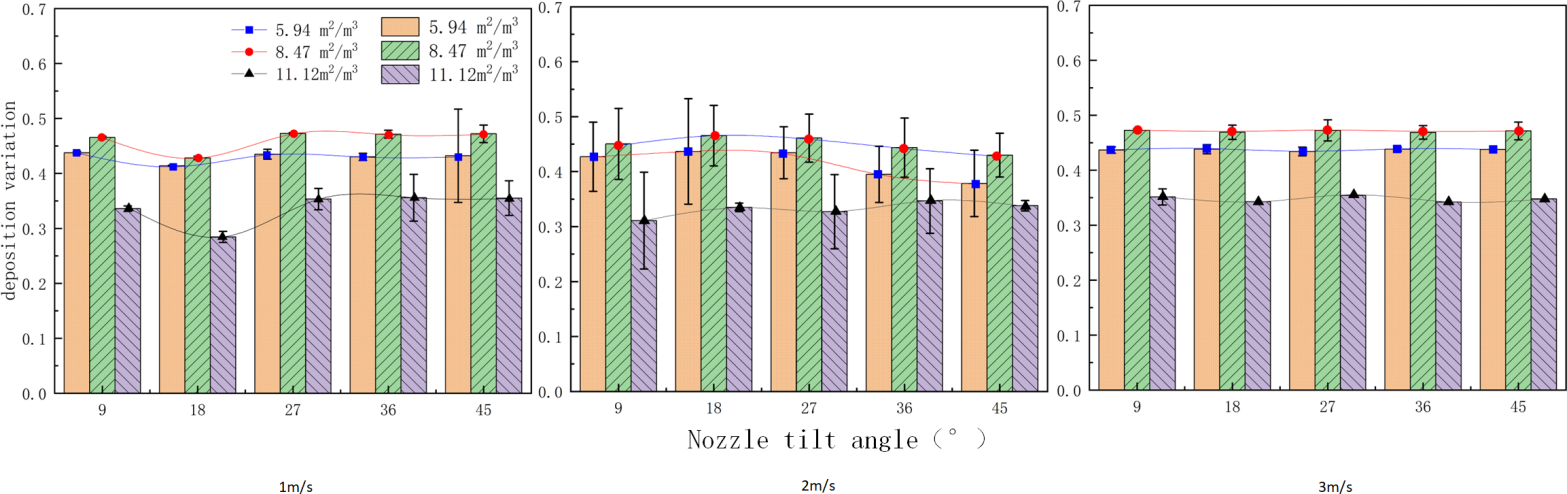
Deposition variation of fog droplets for different parameters.

At a side wind speed of 2 m/s, the difference in the amount of deposited droplets showed an upward parabolic trend with increasing nozzle tilt angle. As shown in **Figure 10**, the minimum deposition difference occurred at a tilt angle of 18°for canopy leaf area densities of 5.94, 8.47, and 11.12 m²/m³, with differences of 0.36, 0.40, and 0.28, respectively. The initial decrease and subsequent increase in droplet deposition volume can be attributed to 1) As side wind speed increases, droplets gain a larger initial velocity, allowing them to penetrate surface leaves more effectively and achieve better penetration. 2) As the nozzle tilt angle increases, the spray direction forms a larger angle with the side wind, resulting in a reverse force that weakens droplet kinetic energy and destabilizes the spray path, reducing penetration effectiveness. 3) At an optimal tilt angle, the side wind and the spray’s initial kinetic energy balance, allowing droplets to enter the canopy along an optimal path, bypassing surface foliage for maximum penetration.

At a side wind speed of 3 m/s, the difference in droplet deposition remained stable with increasing nozzle tilt angle. The deposition difference was constant at 0.43 for a canopy leaf area density of 5.94 m²/m³, 0.47 for 8.47 m²/m³, and 0.35 for 11.12 m²/m³. This stability may result from the saturation of wind speed effects on droplet penetration, stabilizing droplet kinetic energy and movement paths. Canopy density significantly impacts penetration: in low-density canopies, droplets penetrate more effectively; in medium-density canopies, moderate leaf distribution optimizes droplet capture and side wind effects; in high-density canopies, dense foliage blocks droplets, reducing penetration.

In summary, adjusting wind speed and nozzle tilt angle improves droplet penetration in the canopy. Appropriate nozzle tilt angle compensates for droplet drift at different wind speeds, enhancing deposition and penetration, although overall deposition decreases compared to windless vertical spraying. For lower canopy leaf area density (5.94 m²/m³), optimal parameters are a 9° tilt angle and 1 m/s wind speed. For medium canopy leaf area density (8.47 m²/m³), the optimal parameters are an 18° tilt angle and 2 m/s wind speed. For high canopy leaf area density (11.12 m²/m³), the optimal parameters are a 36° tilt angle and 3 m/s wind speed. In practical operations, wind speed and direction vary, and different spray volumes are required for different plant positions (Zhu et al., 2002), necessitating careful selection of the spray nozzle tilt angle.

## 4 Discussion

This paper investigated the average droplet size and spray penetration of horizontal spray under the influence of side winds with varying nozzle inclination angles, focusing on the differences in deposition under different parameters. The following conclusions were obtained:

(1) Under three different wind speeds, the droplet size distribution generally decreases with increasing tilt angle. The maximum droplet size occurred at a 9° tilt angle with a wind speed of 1 m/s, at an 18° tilt angle with a wind speed of 2 m/s, and at an 18° tilt angle with a wind speed of 3 m/s. The highest overall droplet size was observed at a wind speed of 2 m/s and an 18° tilt angle.
(2) The optimal parameters for improving penetration were found to be an 18° tilt angle and a 3 m/s wind speed for a leaf area density of 5.94 m²/m³, a 45° tilt angle and a 2 m/s wind speed for a leaf area density of 8.47 m²/m³, and a 36° tilt angle and a 3 m/s wind speed for a leaf area density of 11.12 m²/m³. Different canopy densities require different optimal parameters, following a certain pattern between nozzle tilt angle and side wind speed.
(3) The amount of droplet deposition on and within the canopy is influenced by side winds. Adjusting the nozzle tilt angle opposite to the wind direction, based on canopy leaf area density, can effectively increase average droplet deposition both inside and outside the canopy. At a wind speed of 1 m/s, the droplet deposition difference followed a downward parabolic trend with increasing tilt angle, reaching maximum values at tilt angles of 9°, 18°, and 36°for different canopy densities. At a wind speed of 2 m/s, the deposition difference followed an upward parabolic trend with increasing tilt angle, reaching a minimum at an 18° tilt angle as canopy density increased. At a wind speed of 3 m/s, the deposition difference did not change with tilt angle but decreased with increasing canopy density.

The novel contributions of this paper encompass the following: The study showed that pesticide penetration in the canopy can be significantly improved by adjusting the nozzle tilt angle and considering side wind speed. This finding has important implications for global agricultural practices, such as integrating droplet prediction models into spraying systems to create “digital twin” for real-time adjustment of optimal parameters, enhancing pesticide use efficiency and crop protection. In resource-limited environments, reducing pesticide use not only lowers agricultural production costs but also minimizes environmental impact, promoting sustainable agricultural development.

Despite the progress made in understanding the effects of side wind speed and nozzle tilt angle on droplet penetration in citrus tree spraying and proposing optimized parameters, limitations remain, particularly regarding the long-term effects. Future research should include long-term field trials to investigate the sustained effects of optimized spray parameters in agricultural production and in-depth studies on the combined effects of wind direction changes, leaf density, air humidity, and tractor speed on spray penetration and efficiency. Further optimization of integrated technology is necessary to provide a comprehensive theoretical and practical basis, especially in the context of global environmental challenges and resource constraints, to promote more efficient and environmentally friendly agricultural production.

## Data availability statement

The raw data supporting the conclusions of this article will be made available by the authors, without undue reservation.

## Author contributions

DS: Conceptualization, Funding acquisition, Investigation, Methodology, Project administration, Resources, Supervision, Validation, Visualization, Writing – original draft, Writing – review & editing. XH: Conceptualization, Data curation, Formal analysis, Investigation, Methodology, Software, Validation, Writing – original draft, Writing – review & editing. JH: Data curation, Software, Writing – original draft. HJ: Data curation, Writing – original draft, Software. SS: Resources, Supervision, Writing – review & editing. XX: Resources, Supervision, Writing – review & editing.
